# Effects of mRNA-VEGF@USPIO magnetic resonance probe on endothelial injury in cerebral aneurysm

**DOI:** 10.3389/fneur.2025.1632617

**Published:** 2025-09-25

**Authors:** Jiang Zhao, Jingyi Li, Haitao Lu, Yalan Chen, Yanming Wang, Xiangyang Liu, Hong Zhao

**Affiliations:** Department of Neurosurgery, Punan Branch of Renji Hospital, Shanghai Jiao Tong University School of Medicine, Shanghai, China

**Keywords:** mRNA-VEGF@USPIO probe, endothelial injury, cerebral aneurysm, MRI, repair

## Abstract

**Objective:**

To investigate the effects of a novel mRNA-VEGF@USPIO magnetic resonance probe on endothelial injury at cerebral aneurysms necks, and to evaluate its imaging performance and therapeutic potential, with a focus on exploring its potential as a therapeutic agent and preliminary imaging characteristics.

**Methods:**

The mRNA-VEGF@USPIO probes were synthesized and thoroughly characterized. A rat model of cerebral aneurysm was successfully established. Vascular morphology, iron deposition, expression of endothelial cells-related factors, and vascular repair processes were evaluated using hematoxylin and eosin (HE) staining, Prussian blue staining, immunohistochemical staining, and magnetic resonance imaging (MRI).

**Results:**

Compared to the model group, both mRNA-VEGF@USPIO probes and rosuvastatin significantly inhibited the proliferation of intimal and medial smooth muscle cells, reduced the risk of luminal thrombosis, and alleviated lumen stenosis. The mRNA-VEGF@USPIO probes additionally promoted the expression of endothelial cell growth-related factors CD31, CD34, VEGF, and vWF. No evidence of iron overload or iron-related toxicity was observed following probe administration. Furthermore, the probes provided high-quality imaging at various concentrations, clearly delineating the location and morphology of the aneurysm neck. Over the treatment course, MRI enabled serial visualization of the progressive recovery process.

**Conclusion:**

The mRNA-VEGF@USPIO probe demonstrated significant efficacy in promoting endothelial repair and regeneration at the neck of cerebral aneurysms. This theranostic agent not only offers a novel treatment strategy for cerebral aneurysms, but its favorable MRI imaging performance also lays a foundation for further evaluating its potential diagnostic value.

## Introduction

Cerebral aneurysm, a prevalent intracranial vascular disorder, affects approximately 2%−5% of the population and poses a significant threat to human health (1). Current primary treatments, including surgical clipping and interventional embolization, are associated with considerable risks and complications, and they exhibit limited efficacy in preventing aneurysm recurrence and re-rupture (2). Therefore, there is a pressing need to develop novel therapeutic strategies and evaluation methods. The pathogenesis and progression of cerebral aneurysm involve multiple factors, with endothelial injury recognized as a central mechanism (3, 4). Such injury leads to structural and functional impairments of the vascular wall, facilitating inflammation, fibrinolysis, apoptosis, and remodeling of the vascular wall, which collectively increase the susceptibility to aneurysm formation and rupture (5, 6). Consequently, protecting or repairing the function of endothelial cells (ECs) represents a promising approach for the prevention and treatment of cerebral aneurysms.

Ultrasmall superparamagnetic iron oxide nanoparticles (USPIO) consist of a magnetite core and a polymeric coating, with an average diameter of ≤ 30 nm, and exhibit superparamagnetic properties (7, 8). These nanoparticles can serve as contrast agents in magnetic resonance imaging (MRI) by altering T1 and T2 relaxation times in surrounding tissues or blood, thereby inducing significant signal enhancement (9, 10). USPIO possess several advantageous characteristics, including good biocompatibility, pro-longed circulation half-life, high tissue specificity, ease of functionalization, rendering them highly suitable for various applications in medical diagnosis and treatment (11, 12). For instance, USPIO have been utilized for contrast-enhanced imaging of reticuloendothelial system-rich organs, such as liver, spleen, lymph nodes, bone marrow, as well as for vascular and even coronary imaging (13, 14). Furthermore, through conjugation with specific ligands or antibodies, USPIO enable targeted cellular labeling and tracking, allowing precise identification and imaging of particular cells or molecules (15).

Vascular endothelial growth factor (VEGF) is a crucial angiogenic factor, involved in vascular formation and repair in various physiological and pathological processes (16). By binding to and activating VEGF2, VEGF stimulate endothelial cell proliferation, migration, differentiation and survival, thereby accelerating the repair of damaged endothelium (17, 18). In addition, studies have shown that VEGF VEGF expression is subject to complex and coordinated regulation during endothelial injury (19, 20). These properties make VEGF a promising therapeutic candidate for repairing cerebral microvascular endothelial damage. However, conventional drug delivery approaches lack the specificity to direct exogenous VEGF to target tissues, substantially limiting its therapeutic efficacy. The use of nanoparticle-based carriers enables precise and targeted delivery of therapeutic agents to specific tissues or organs, enhancing treatment outcomes. Based on this rationale, the present study developed a novel mRNA-VEGF@USPIO magnetic probe and evaluated its application for *in vivo* imaging and treatment in a rat model of carotid artery balloon injury.

## Methods

### Animal model establishment and grouping

A total of thirty-six 4-week-old male Sprague-Dawley (SD) rats weighing 350–400 g were purchased from Shanghai SLAC Laboratory Animal Co., Ltd. (Shanghai, China). All animal procedures were conducted in compliance with the ARRIVE guidelines and approved by the Ethics Committee Renji Hospital, Shanghai Jiaotong University School of Medicine (Approval number: 2023-539). Additionally, the mRNA-VEGF@USPIO probes (zeta potential: about −0.3 mV, favoring neutral; primary particle size peak: 80.1 nm) used in this exploration have been successfully prepared and characterized in our previous study (21).

The rats were randomly divided into six groups, including control, model, low-dose probe, medium-dose probe, high-dose probe, and rosuvastatin groups, with six male SD rats in each group. Except for the control group, the rats in the other groups were employed to construct a carotid artery balloon injury model as previously described (22). Briefly, after anesthesia with 10% sodium barbital, the left common, external and internal carotid arteries were exposed in turn. Then, the distal end of the external carotid artery was ligated and the common and internal carotid arteries were temporarily clamped. The common carotid artery was released and a 2F balloon catheter was inserted through the incised external carotid artery into the aortic arch, and 0.1–0.2 ml of saline was injected into the balloon. Then, the catheter was slowly pulled outwards, and this procedure was repeated three times before withdrawing the catheter and temporarily clamping the common carotid artery. The catheter was removed and the proximal end of the incised external carotid artery was ligated, and the clamps on the common and internal carotid arteries were released to restore blood flow. The skin incision was sutured. After the operation, 400,000 units of penicillin were administered by intramuscular injection to prevent infection.

After establishing the rat model, 400 U of heparin was intravenously injected into the rats for three consecutive days. It was obvious that the initial vascular injury degree (such as endothelial shedding area, basal level of inflammatory factors) of rats in each group after modeling was uniform due to the same batch. After that, the rats in the model, low-dose probe, medium-dose probe, and high-dose probe groups were subcutaneously injected with saline, 10 μg/mL of mRNA-VEGF@USPIO probes, 25μg/mL of mRNA-VEGF@USPIO probes and 50 μg/mL of mRNA-VEGF@USPIO probes (23), respectively, once a week. However, the rats in the rosuvastatin group were administered with 20 mg/kg/d rosuvastatin by gavage as a positive control group; whereas the rats in the control group were without any treatments. After 20 weeks of continuous administration, the treatment was stopped, and the rats were anesthetized with 10% sodium barbital and euthanized. The vascular specimens were collected, and some were fixed in 4% paraformaldehyde solution for pathological histological analysis, while others were stored at −80 °C for subsequent experiments.

### Hematoxylin and eosin (HE) staining

Vascular tissues from different groups were fixed in 4% paraformaldehyde, and subsequently dehydrated through an automatic ethanol-xylene series as follows: 70% ethanol for 3 h, 80% ethanol for 45 min, 95% ethanol I for 50 min, 95% ethanol II for 50 min, absolute ethanol I for 40 min, absolute ethanol II for 40 min, xylene I for 1 h, xylene II for 1 h). After paraffin embedding, the vascular tissue samples were cut into 4 μm thick sections. After deparaffinization and rehydration, the sections were stained with hematoxylin (BASO, BA-4097, China) for 10 min, and differentiated in 1% hydrochloric acid alcohol for 3 s, and then counterstained with eosin (BASO, BA-4099) for 90 s. Fianlly, the sections were mounted with neutral gum, and examined under an inverted microscope (IX70, Olympus Corporation, Japan) for imaging.

### Prussian blue staining

Paraffin sections were dewaxed and rehydrated to distilled water. After three washes in distilled water, sections were treated with a mixture of 5 wt% potassium ferrocyanide and 10 vol% hydrochloric acid for 15 min at room temperature. Following another three washes in distilled water, nuclei were counterstained with nuclear fast red solution for 3 min. Stained sections were imaged under an inverted microscope (IX70, Olympus Corporation, Japan).

### Immunohistochemical staining

Deparaffinized and rehydrated sections were subjected to antigen retrieval by autoclaving in citrate buffer (pH 6.0) for 10 min. Endogenous peroxidase activity was quenched with 3% H_2_O_2_ for 15 min at room temperature. After blocking with goat serum for 1 h at 37 °C, sections were incubated overnight at 4 °C with the following primary antibodies: anti-CD31 (Abcam ab182981, 1:2,000), anti-CD34 (Abcam ab81289, 1:2,000), anti-vWF (Servicebio GB11020, 1:500), and anti-VEGF (Proteintech 9003-1-AP, 1:100). After washing three times with PBST, sections were incubated with horseradish peroxidase (HRP)-conjugated goat anti-mouse IgG secondary antibody (Jackson ImmunoResearch, 115-035-003) for 30 min at 37 °C. Signal detection was performed using 3,3′-diaminobenzidine (DAB; Beijing Zhongshan Jinqiao Biotechnology). Finally, sections were counterstained with hematoxylin, dehydrated through an ethanol-xylene series, and mounted.

### Magnetic resonance imaging

At 4, 12, and 24 weeks after mRNA-VEGF@USPIO probe administration, rats were anesthetized via intravenous injection of 10% chloral hydrate, positioned prone, and secured using a dedicated head coil. Aneurysmal vessels were imaged using a 3.0T MRI scanner with a T2-weighted fast spin-echo sequence (TR: 3,000 ms; TE: 90.4 ms; slice thickness: 1 mm; slice gap: 1 mm; matrix: 256 × 256; FOV: 8 cm × 8 cm). For signal intensity analysis, regions of interest (ROIs >30 mm^2^) were placed in consistent cross-sectional locations. Each sample was measured three times, and results are expressed as mean ± standard deviation.

### Statistical analysis

All experiments were performed in triplicate. Data are presented as mean ± standard deviation (SD). Differences between two groups were assessed using Student's *t*-test, and multiple-group comparisons were analyzed by one-way ANOVA with SPSS 19.0. Graphs were generated using GraphPad Prism 5. A *p-*value < 0.05 was considered statistically significant.

## Results

### Effects of mRNA-VEGF@USPIO probe treatment on the physiological morphology of blood vessels

**Figure 1:**
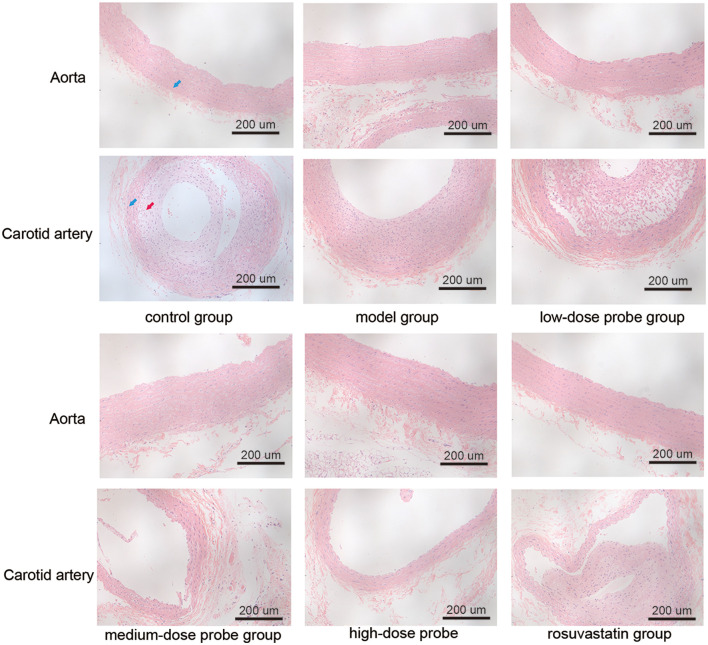
Hematoxylin and eosin staining of aorta and carotid artery of rats in different treatment groups at the magnification time of 200×. The **blue arrow** indicates the tunica media, and the **red arrow** indicates the tunica intima.

To evaluate the impact of mRNA-VEGF@USPIO probe and drug treatment on vascular morphology, HE staining was performed (Figure 1). Under the light microscope, aortic and carotid artery sections in the control rats exhibited sparse endothelial cells and a well-defined elastic layer structure. However, in the model rats (arterial vascular injury using balloons), distinct endothelium was seen in tissue sections of aorta and carotid artery, with homogeneous or heterogeneous hyperplasia, and centripetal or eccentric narrowing of the lumen, as well as hyperplasia of smooth muscle cells were seen in the endothelium, with a small accumulation of intercellular interstitium and varying degrees of disruption of the internal elastic plate. Furthermore, at the breaks, the mesothelium was connected to the endothelial hyperplastic smooth muscle cells, and there was a narrowing of the luminal canal. Compared to the model rats, the rats treated with low, medium, and high concentration probes (low-, medium-, and high-dose probe groups), as well as those receiving rosuvastatin (rosuvastatin group) showed a noticeable reduction in the proliferation of intimal and medial smooth muscle cells, decreased risk of luminal thrombosis, and attenuated lumen narrowing. These results indicate that mRNA-VEGF@USPIO probes exert restorative effects on vascular endothelial injury comparable to those of rosuvastatin. Moreover, the beneficial impact of mRNA-VEGF@USPIO probes appears to be consistent across different concentrations, indicating that the therapeutic effect is not dose-dependent within the tested range.

### Prussian blue iron staining of vascular tissues

**Figure 2:**
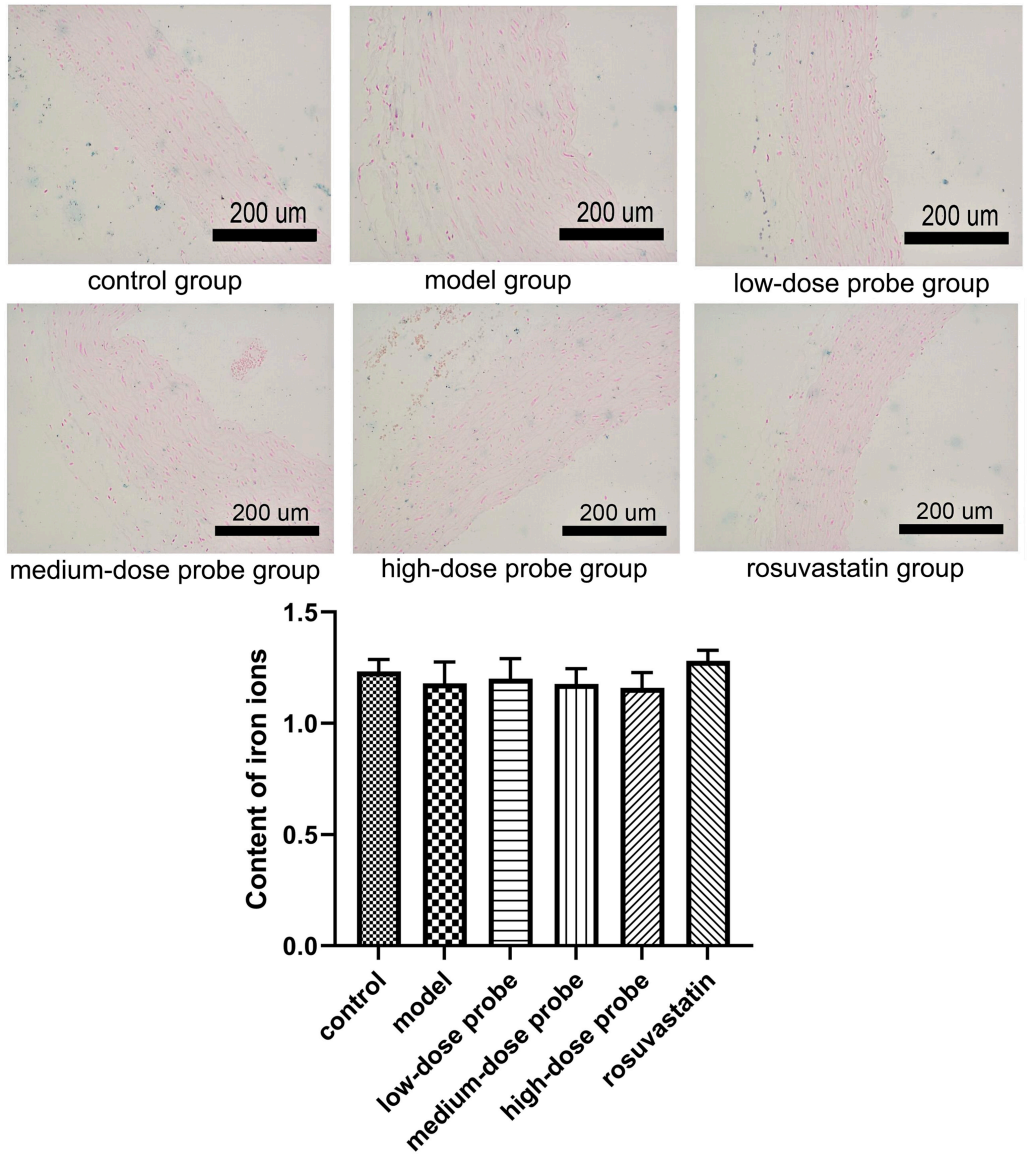
Prussian blue iron staining of rat aortic sections in different treatment groups. There was no significant difference in iron ions among the different groups (*P* > 0.05).

Subsequently, Prussian blue staining was performed to detect ferric iron deposition in rat aortic sections. The results revealed mild iron accumulation in the aortic tissues across all experimental groups. However, no significant differences in iron content were observed among the control, model, low-dose probe, medium-dose probe, high-dose probe, and rosuvastatin groups (*P* > 0.05, Figure 2).

### Immunohistochemistry of the aorta in rats

**Figure 3:**
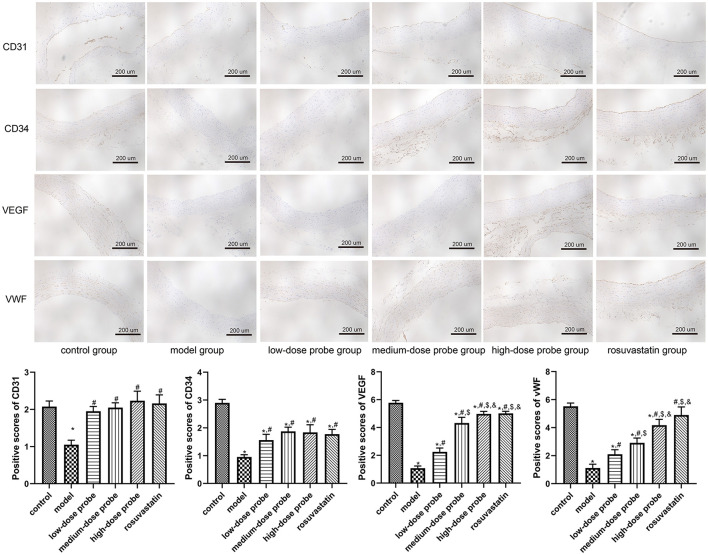
Protein expression of CD31, CD34, VEGF, and vWF in the rat aortic sections examined using immunohistochemical staining (200×). *P < 0.05, vs. control group; ^#^*P* < 0.05, vs. model group; ^$^*P* < 0.05, vs. low-dose probe group; ^&^*P* < 0.05, vs. medium-dose probe group.

Further, immunohistochemical staining was employed to evaluate the expression of CD31, CD34, VEGF, and vWF in rat aortic sections. The results demonstrated that, compared with the control group, the protein levels of CD31, CD34, VEGF, and vWF were significantly downregulated in the model group (*P* < 0.05). In contrast, administration of low-, medium-, and high-dose mRNA-VEGF@USPIO probes, as well as rosuvastatin gavage, markedly elevated the expression of these endothelial markers relative to the model group (*P* < 0.05; Figure 3). These findings indicate that both mRNA-VEGF@USPIO probes and rosuvastatin effectively promoted the expression of endothelial cell growth-related factors, thereby facilitating endothelial repair.

### MRI for aneurysm angiography

**Figure 4:**
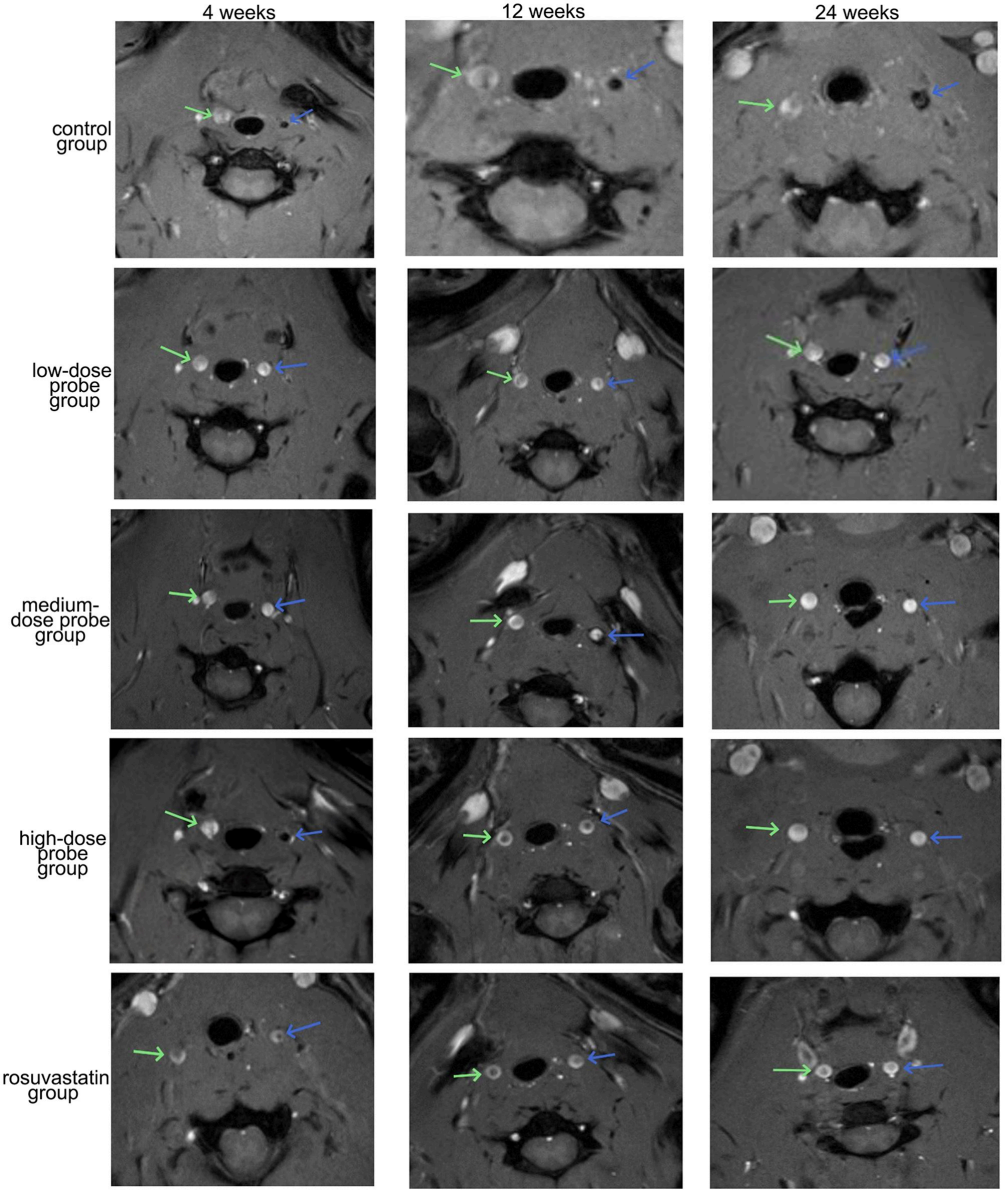
Representative images of MRI detection of aneurysm angiography. The **blue arrow** indicates the left carotid artery, and the **green arrow** indicates the right carotid artery.

MRI imaging of the aneurysmal vessels was performed at the right wall, right tube, left arm, and left tube at 4, 12, and 24 weeks following the administration of different concentrations of mRNA-VEGF@USPIO probes or the gavage of rosuvastatin (Figure 4). The results indicated that the enhancement degree of the MRI imaging was similar at the same site with different concentrations of mRNA-VEGF@USPIO probe at 4, 12, and 24 weeks. In order to further quantitatively observe the repair process of endothelial cells after injury, the RediAnt 2023.1 software was used to measure the signal intensity of the carotid artery lumen and the enhanced ring on the inner wall of the carotid artery in the MRI images. It was found that the concentration of the probes had no significant effects on the MRI contrast results (Table 1). It is speculated that in clinical practice, a lower concentration of mRNA-VEGF@USPIO probes may be sufficient to promote the regeneration of endothelial cells and real-time imaging tracking, and can also avoid the side effects that may be caused by high-concentration probes. In addition, with the extension of treatment time, mRNA-VEGF@USPIO probe not only did not damage the repaired vessels, but also promoted the growth of the aneurysmal neck vessels, and could show their gradual recovery process.

**Table 1:** The signal intensity measurement results of MRI vascular imaging for the right lumen, right wall, left lumen, and left wall.

**Weeks**	**Group**	**Right lumen**	**Right wall**	**Left lumen**	**Left wall**
4	Control	2.58 ± 0.77	3.26 ± 0.51	2.65 ± 0.13	2.92 ± 0.06
	Low-dose	2.70 ± 1.00	3.11 ± 0.31	2.53 ± 0.41	2.64 ± 0.97
	Medium-dose	3.03 ± 0.14	3.73 ± 0.49	2.96 ± 0.74	3.09 ± 0.53
	High-dose	2.70 ± 0.31	3.47 ± 0.09	2.47 ± 0.24	2.86 ± 0.57
12	Control	2.26 ± 0.76	3.08 ± 0.53	2.43 ± 0.21	2.927 ± 0.59
	Low-dose	2.28 ± 0.80	3.18 ± 0.59	2.56 ± 1.02	2.81 ± 1.00
	Medium-dose	2.16 ± 0.35	3.16 ± 0.14	2.84 ± 1.22	3.43 ± 1.21
	High-dose	2.34 ± 0.58	3.31 ± 0.44	2.28 ± 0.37	3.21 ± 0.75
24	Control	3.00 ± 0.76	3.40 ± 0.50	2.54 ± 0.03	4.4 ± 1.02
	Low-dose	2.98 ± 1.00	3.91 ± 0.33	2.48 ± 0.83	4.28 ± 0.66
	Medium-dose	3.03 ± 0.16	3.73 ± 0.51	2.83 ± 0.59	4.32 ± 0.54
	High-dose	3.01 ± 0.28	3.48 ± 0.14	2.93 ± 0.22	4.84 ± 0.36

Data were expressed mean ± standard deviation. The signal intensity value has no absolute physical unit, which is usually presented as a grayscale value or relative signal intensity (RSI). It is used to reflect the difference in the strength of the magnetic resonance signals of tissues.

## Discussion

Cerebral aneurysm is a common intracranial vascular abnormality that can lead to severe clinical outcomes, including subarachnoid hemorrhage, cerebral infarction, and neurological dysfunction, posing a significant threat to patient health and survail (24, 25). Epidemiological studies indicate that approximately 3%−5% of the global population harbors cerebral aneurysms, with an annual rupture rate of 10%−15% among affected individuals (26, 27). The pathogenesis and progression of cerebral aneurysms are related to various factors, such as genetic factors, hemodynamic factors, endothelial cell injury, inflammatory response, and vascular wall structure abnormalities (28, 29). At present, the treatment of cerebral aneurysms mainly includes surgical clipping and interventional embolization, but both have certain risks and complications, such as rebleeding, thrombosis, vascular stenosis (30, 31). Consequently, the search for new treatment strategies is important to improve the outcome and prognosis of cerebral aneurysms. This study aimed to investigate the efficacy of a novel mRNA-VEGF@USPIO magnetic resonance probe in mitigating endothelial injury at the neck of cerebral aneurysms.

Our results demonstrated that, compared to the model group, both the mRNA-VEGF@USPIO magnetic resonance probe and rosuvastatin could significantly reduce the proliferation of intimal and medial smooth muscle cells, decreased the risk of luminal thrombosis, and attenuated lumen stenosis. These findings suggest that both interventions confer restorative effects on vascular endothelial injury. In addition, treatment with the mRNA-VEGF@USPIO magnetic resonance probe or rosuvastatin can markedly up-regulate the expression of CD31, CD34, VEGF and vWF related to endothelial cell growth, which are beneficial to endothelial cell repair. CD31 and CD34 are endothelial cell-specific markers implicated in angiogenesis, vascular permeability, cell adhesion and migration processes (32, 33). VEGF is a potent pro-angiogenic cytokine that can stimulate endothelial cell proliferation, migration and differentiation (34), while vWF, secreted by endothelial cells, mediates platelet adhesion and contributes to hemostasis and coagulation (35). The observed downregulation of these factors in the cerebral aneurysm model group and their subsequent upregulation following treatment with either the mRNA-VEGF@USPIO probe or rosuvastatin imply their involvement in the pathogenesis and progression of cerebral aneurysms. We hypothesize that the mRNA-VEGF@USPIO probe may facilitate VEGF gene expression in injured endothelial cells, thereby activating downstream VEGF signaling cascades that promote endothelial regeneration, vascular remodeling, and enhanced vascular integrity. The precise molecular mechanisms underlying these effects warrant further investigation.

In addition to its therapeutic benefits, the mRNA-VEGF@USPIO probe exhibited strong magnetic resonance signal enhancement, allowing clear visualization of cerebral aneurysm morphology and post-treatment changes. Probe concentration had minimal impact on MRI outcomes, suggesting that even lower concentrations may suffice for clinical imaging, thereby reducing potential side effects. Moreover, prolonged treatment with the probe not only avoided detrimental effects on repaired vessels but also promoted vascular regrowth at the aneurysm neck, enabling non-invasive monitoring of the recovery process over time. This theranostic capability, combining therapeutic efficacy and diagnostic imaging, highlights the potential of the mRNA-VEGF@USPIO probe as a valuable tool in the clinical management of cerebral aneurysms.

Finally, mRNA-VEGF@USPIO magnetic resonance probe induced only minimal iron deposition in rat aortic tissues, with no significant differences among experimental groups, indicating favorable biocompatibility and *in vivo* safety. USPIO is a superparamagnetic iron oxide nanoparticle that can act as a contrast agent for magnetic resonance imaging enhancing signal (36). However, USPIO may also cause iron ion deposition and toxicity leading to tissue damage. Our results showed that mRNA-VEGF@USPIO magnetic resonance probe has no obvious iron ion deposition in rat aortic tissue and there is no difference from normal control group indicating that its distribution and clearance *in vivo* are uniform without causing local iron overload or toxic reaction.

The consistent therapeutic efficacy and imaging performance across low-, medium-, and high-dose mRNA-VEGF@USPIO groups, validated by quantitative analyses, merit discussion. One-way ANOVA confirmed no significant differences between dose groups in key indices: endothelial marker expression (CD31, CD34, VEGF, vWF), and MRI signal intensity. Several factors may underlie this dose independence.

Firstly, the low-dose probe (10 μg/mL) likely reaches the saturation threshold for VEGF-mediated effects. VEGF exhibits a bell-shaped dose-response in vascular repair (37), with excess concentrations failing to enhance efficacy; our data suggest the low dose already achieves optimal endothelial regeneration, making higher doses redundant. Secondly, USPIO's biological distribution contributes: its small size (80.1 nm) and near-neutral zeta potential (−0.3 mV) enable targeted accumulation at injury sites via the Enhanced Permeability and Retention (EPR) effect (38). Above a certain dose, EPR-mediated targeting plateaus, limiting further increases in local concentration—consistent with reports that USPIO accumulation in damaged vasculature stabilizes within a narrow range (39). Thirdly, quantitative Prussian blue staining (ANOVA, *P* > 0.05) showed no dose-related differences in iron deposition, indicating efficient clearance of excess iron via physiological pathways (40), preventing toxicity even at high doses. Clinically, this dose independence is advantageous: low-dose probes suffice for therapeutic (endothelial repair) and diagnostic (MRI) purposes, reducing systemic risks and costs. Future studies will explore sub-10 μg/mL ranges to define the minimal effective dose, optimizing safety and feasibility.

Human cerebral aneurysms, typically saccular protrusions driven by hemodynamic stress, are characterized by localized vascular wall weakening, aberrant flow patterns (e.g., elevated wall shear stress, recirculation zones), and progressive structural deterioration of the aneurysm neck and dome (41, 42). In contrast, the carotid artery balloon injury model primarily recapitulates endothelial denudation, followed by intimal hyperplasia, smooth muscle cell proliferation, and luminal stenosis—pathological processes more aligned with post-injury vascular remodeling than saccular aneurysm formation (43). These distinctions, including the lack of saccular outpouching and divergent hemodynamic triggers, constitute key limitations of our current model (44). Notably, however, endothelial injury represents a critical shared mechanism. In human cerebral aneurysms, endothelial dysfunction initiates a cascade involving impaired vascular integrity, inflammatory infiltration, and aberrant smooth muscle cell activation, all of which heighten rupture risk (45, 46). Similarly, the balloon injury model induces endothelial damage that recapitulates core vascular repair processes (e.g., endothelial regeneration, smooth muscle cell modulation) pivotal for stabilizing the aneurysm neck (47). Thus, despite differing overall pathologies, the model effectively captures the endothelial injury-repair axis central to aneurysm pathophysiology. Our findings demonstrate that the mRNA-VEGF@USPIO probe promotes endothelial regeneration (via upregulating CD31, CD34, VEGF, and vWF) and mitigates excessive smooth muscle cell proliferation—effects relevant to aneurysm neck stabilization, where intact endothelium and balanced remodeling are critical for preventing rupture (48). Additionally, the probe's capacity to visualize repair processes establishes a basis for future studies in more clinically representative aneurysm models.

However, there are some limitations in this study. First, we acknowledge that verifying the probe's efficacy in a hemodynamically induced saccular aneurysm model (e.g., rodent models with intracranial aneurysm induction via hypertension or elastase infusion) and complementing with computational fluid dynamics analysis (to assess interactions with aneurysm-specific flow patterns) would strengthen clinical translatability. Second, subsequent experiments will be designed to study the imaging performance of mRNA-VEGF@USPIO probes in comparison with other contrast agents, as well as to evaluate the biological safety of mRNA-VEGF@USPIO probes (including systemic toxicity, local tissue inflammation or immune response, potential impact of long-term residues on the body, etc.). Additionally, the iron ion deposition in other major organs should be determined in the future; as well as future studies will specifically compare mRNA-VEGF@USPIO with VEGF-free USPIO probes, focusing on evaluating their imaging specificity, sensitivity, and correlation with pathological changes in cerebral aneurysms.

## Conclusion

In summary, this study confirmed that the novel mRNA-VEGF@USPIO magnetic resonance probe had significant repair effects on endothelial injury in the neck of cerebral aneurysms, with favorable imaging performance that supports further exploration of its diagnostic potential. It provides a new strategy for the treatment of cerebral aneurysms, and subsequent studies will focus on evaluating its imaging properties to achieve the goal of developing a theranostic agent.

## Data Availability

The original contributions presented in the study are included in the article/supplementary material, further inquiries can be directed to the corresponding author.
